# Dysregulation of peritoneal cavity B1a cells and murine primary biliary cholangitis

**DOI:** 10.18632/oncotarget.8853

**Published:** 2016-04-20

**Authors:** Yan-Qing Yang, Wei Yang, Yuan Yao, Hong-Di Ma, Yin-Hu Wang, Liang Li, Qingfa Wu, M. Eric Gershwin, Zhe-Xiong Lian

**Affiliations:** ^1^ Liver Immunology Laboratory, Institute of Immunology and The CAS Key Laboratory of Innate Immunity and Chronic Disease, School of Life Sciences, University of Science and Technology of China, Hefei, China; ^2^ The CAS Key Laboratory of Innate Immunity and Chronic Disease, School of Life Sciences, University of Science and Technology of China, Hefei, China; ^3^ Division of Rheumatology, Allergy and Clinical Immunology, University of California at Davis School of Medicine, Davis, CA, United States of America; ^4^ Innovation Center for Cell Signaling Network, Hefei National Laboratory for Physical Sciences at Microscale, Hefei, China

**Keywords:** B1a cell, autoimmune cholangitis, dysregulation, Breg, peritoneal cavity, Immunology and Microbiology Section, Immune response, Immunity

## Abstract

Primary biliary cholangitis (PBC) is a chronic autoimmune liver disease with progressive cholestasis and liver fibrosis. Similar to human patients with PBC, *p40^−/−^IL-2Rα^−/−^* mice spontaneously develop severe autoimmune cholangitis. Although there has been considerable work on immune regulation and autoimmunity, there is a relative paucity of work directed at the functional implications of the key peritoneal cavity (PC) B cell subset, coined B1a cells in PBC. We used flow cytometry and high-resolution microarrays to study the qualitative and quantitative characteristics of B cells, particularly B1a cells, in the PC of *p40^−/−^IL-2Rα^−/−^* mice compared to controls. Importantly, B1a cell proliferation was markedly lower as the expression of Ki67 decreased. Meanwhile, the apoptosis level was much higher. These lead to a reduction of B1a cells in the PC of *p40^−/−^IL-2Rα^−/−^* mice compared to controls. In contrast, there was a dramatic increase of CD4^+^ and CD8^+^ T cells accompanied by elevated production of IFN-γ. In addition, we found a negative correlation between the frequency of B1a cells and the presence of autoreactive CD8^+^ T cells in both liver and PC of *p40^−/−^IL-2Rα^−/−^* mice. From a functional perspective, B cells from *p40^−/−^IL-2Rα^−/−^* mice downregulated IL-10 production and CTLA-4 expression, leading to loss of B cell regulatory function. We suggest that the dysfunction of B1a cells in the PC in this murine model of autoimmune cholangitis results in defective regulatory function. This highlights a new potential therapeutic target in PBC.

## INTRODUCTION

It is interesting that the liver, whose immunological function is to facilitate immune tolerance, itself becomes a victim of autoimmunity, including primary biliary cholangitis (PBC), a disease characterized by lymphocytic infiltrates in portal tracts and the presence of anti-mitochondrial Abs (AMAs) secreted by autoreactive B cells [1–7]. A major difficulty in understanding PBC is that the latency time between the onset of autoantibodies and the appearance of clinical disease may lag by years. Thus the use of murine models has become particularly important in defining the earliest events that lead to portal inflammation [8–12].

An interesting feature of both humans and mice with autoimmune cholangitis is that although there are high titer AMAs, the role of B cells in the pathogenesis of disease has remained enigmatic [13–16]. Previous work on B cells in a variety of murine models of autoimmunity have emphasized that there are two distinct B cell lineages, including B1 (including B1a and B1b) and B2. These populations arise from distinct progenitors and differ in development, tissue distribution, phenotype and function [17–19]. Importantly, B cell populations include a regulatory subpopulation whose function and clinical importance continues to be defined [20–24]. In addition, there is a unique B cell subpopulation, namely the B1 cells, is enriched in the PC. This subpopulation contributes to immune regulation through spontaneous production of natural antibodies, and can function as an antigen-presenting cell [25–33]. Our laboratory has focused on a unique model of PBC, *p40^−/−^IL-2Rα^−/−^* mice [12]. This model not only manifests severe portal inflammation/bile duct damage, but also develops liver fibrosis. We have focused on the role of B1 cells in this model and report herein a contribution of B1a cell dysfunction to the loss of tolerance by alteration of regulatory pathways. These data take on significance not only for PBC, but also focus in further defining the mechanisms of immune tolerance and B1 subpopulations.

## RESULTS

### Quantitation of PC subsets

**Figure 1:**
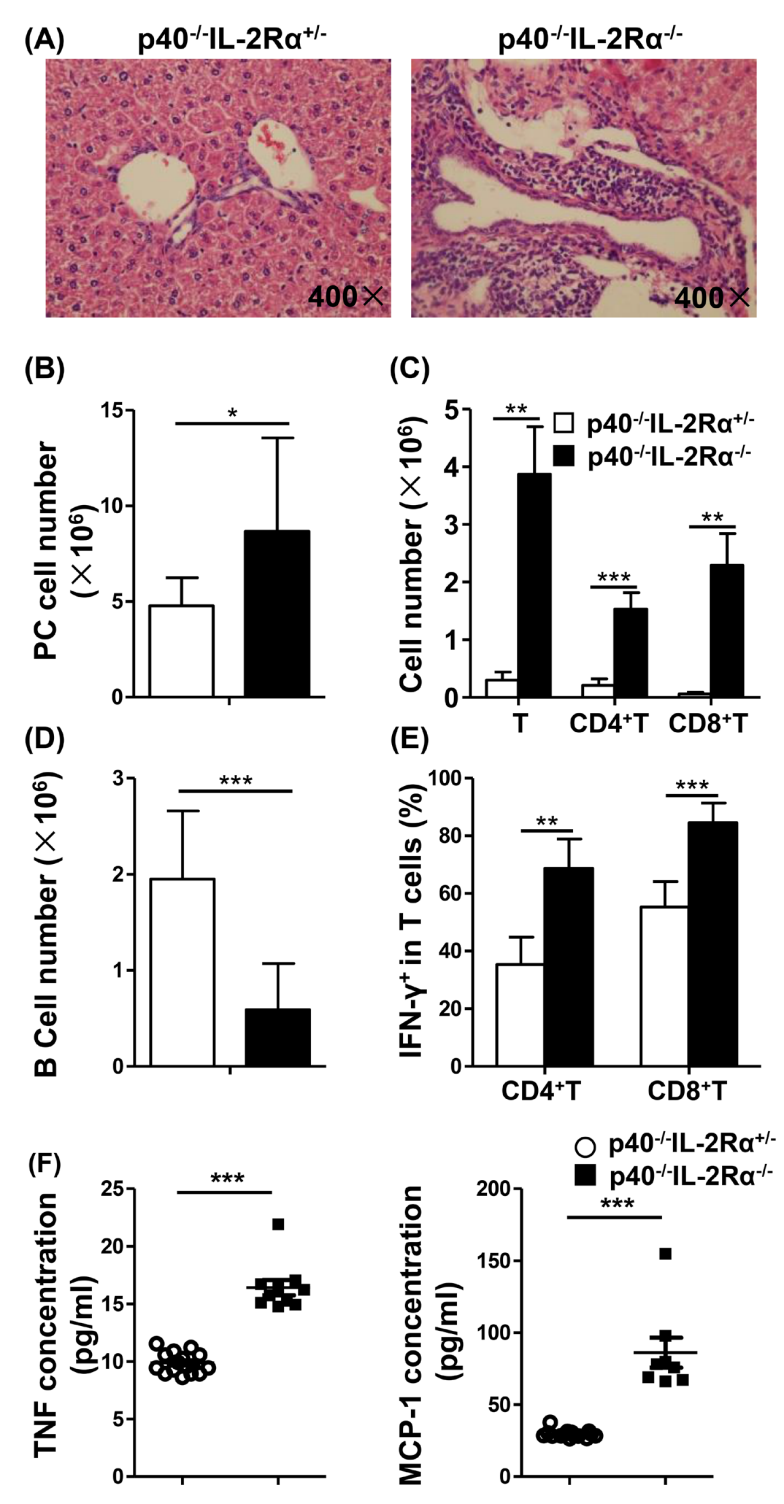
There was a decrease of B cells, an increase of total cells, including T cells, in the PC of *p40^−/−^IL-2Rα^−/−^* mice **A.** H&E staining of liver sections from *p40^−/−^IL-2Rα^−/−^* mice and *p40^−/−^IL-2Rα^+/−^* mice. **B.** Numbers of total cells in the PC of *p40^−/−^IL-2Rα^−/−^* (*n* = 13) and *p40^−/−^IL-2Rα^+/−^* mice (*n* = 9). Total number of T cells, CD4^+^ T cells, CD8^+^ T cells **C.** and B cells **D.** in the PC of *p40^−/−^IL-2Rα^−/−^* mice (*n* = 5) and *p40^−/−^IL-2Rα^+/−^* mice (*n* = 9). **E.** The frequency of IFN-γ^+^ cells gated on CD4^+^ and CD8^+^ T cells in PC of *p40^−/−^IL-2Rα^−/−^* mice (*n* = 5) and *p40^−/−^IL-2Rα^+/−^* mice (*n* = 4). **F.** PC lavage fluid cytokine levels of *p40^−/−^IL-2Rα^−/−^* mice (*n* = 8) and *p40^−/−^IL-2Rα^+/−^* mice (*n* = 15). *P < 0.05, **P < 0.01, ***P < 0.001.

As expected, and for the purpose of control only, we noted significant portal infiltrates and bile duct injury in the liver of 12 week old *p40^−/−^IL-2Rα^−/−^* mice (Figure 1A). Total number of PC cells was markedly increased in *p40^−/−^IL-2Rα^−/−^* mice, compared to *p40^−/−^IL-2Rα^+/−^* mice (*P* = 0.0216, Figure 1B and Table 1). The numbers of T cells (*P* = 0.0015), CD4^+^ T cells (*P* = 0.0008) and CD8^+^ T cells (*P* = 0.0024) were much higher in PC of *p40^−/−^IL-2Rα^−/−^* compared to *p40^−/−^IL-2Rα^+/−^* mice, while B cell number (*P* < 0.0001) was dramatically lower (Figure 1C, 1D and Table 1). In PC CD4^+^ and CD8^+^ T cells, Th1 cell associated cytokine IFN-γ was higher in *p40^−/−^IL-2Rα^−/−^* mice compared to *p40^−/−^IL-2Rα^+/−^* controls (P = 0.002 & *P* < 0.0001) (Figure 1E). As noted earlier, we initially compared control mice with 3 genotypes and found them similar in liver histology, cell number and cytokine secretion (Figure S1). Thence we used littermate *p40^−/−^IL-2Rα^+/−^* mice as controls throughout these studies. We thought that the change of PC cell subsets in *p40^−/−^IL-2Rα^−/−^* mice might be resulted from the inflammatory environment of PC. To support our hypothesis, we analyzed the level of inflammatory cytokines in PC. Importantly, the concentrations of TNF and MCP-1 were significantly increased in PC lavage fluid of *p40^−/−^IL-2Rα^−/−^* mice compared to *p40^−/−^IL-2Rα^+/−^* mice (*P* < 0.0001 & *P* < 0.0001, Figure 1F). These data showed a significant quantitative difference in the PC subpopulations of *p40^−/−^IL-2Rα^−/−^* mice.

**Table 1:** Cell number of immune cell subsets in the peritoneal cavity

	*p40^−/−^IL-2Rα^+/−^*	*p40^−/−^IL-2Rα^−/−^*
Total cell number (n×10^6^)	4.78±1.46	7.58±3.10*
T cell	0.30±0.14	3.87±2.86**
CD4^+^ T cell	0.21±0.11	1.53±0.99***
CD8^+^ T cell	0.06±0.01	2.29±1.91**
B cell	1.95±0.71	0.59±0.48***
NK cell	0.07±0.06	0.02±0.02*
Macrophage	2.04±0.75	2.23±0.61

* *P* < 0.05;

** *P* < 0.01;

*** *P* < 0.001, compared with *p40^−/−^IL-2Rα^+/−^* mice.

### Correlation of portal inflammation and B1a cell frequency

**Figure 2:**
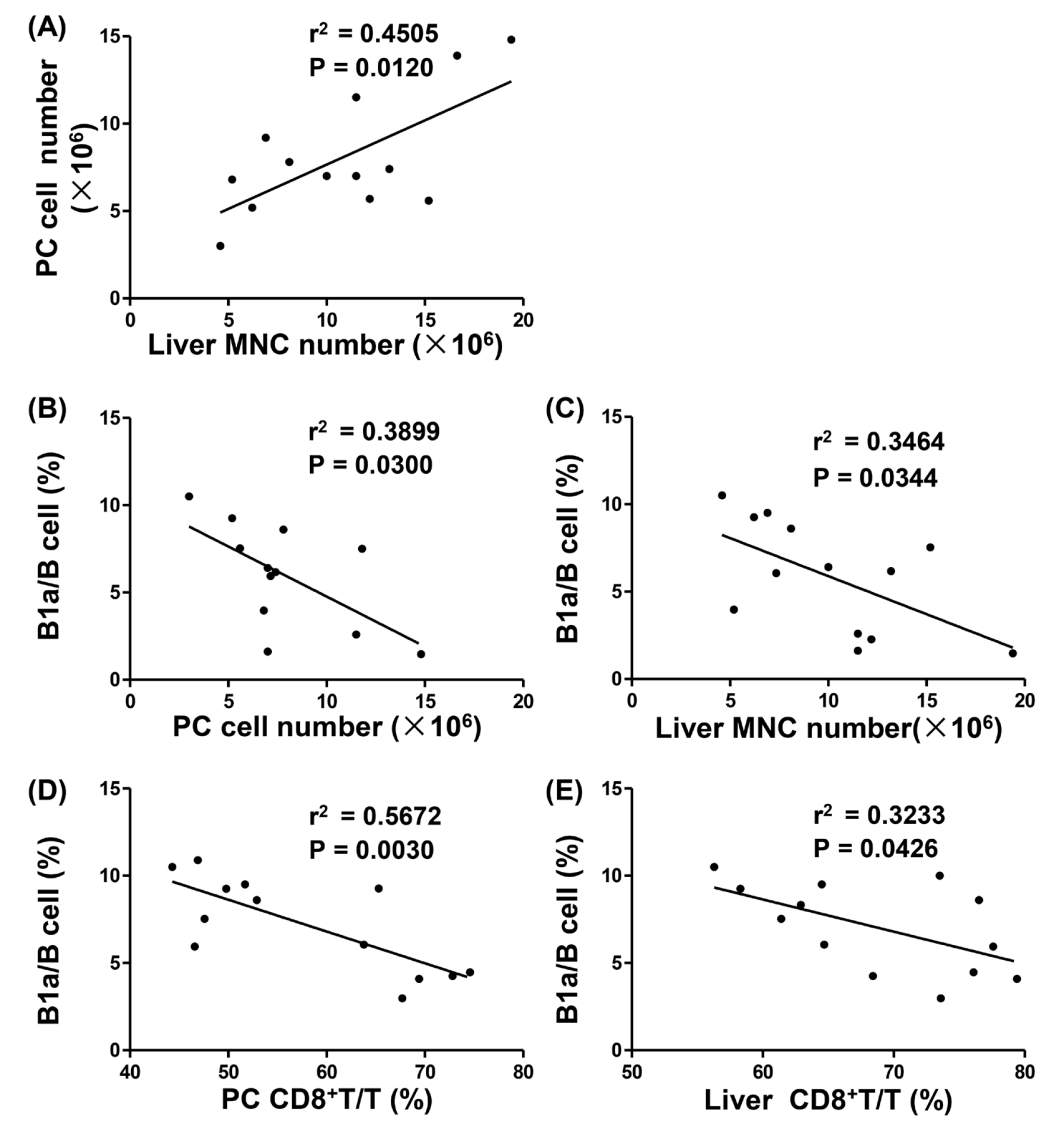
Correlation between liver inflammation and PC B1a cells in *p40^−/−^IL-2Rα^−/−^* mice **A.** Linear regression analysis between PC cell numbers and liver MNC numbers of *p40^−/−^IL-2Rα^−/−^* mice (*n* = 13). Linear regression analysis between the ratio of B1a/B cells and cell numbers in PC (*n* = 12) **B.** and liver (*n* = 13) **C.** of *p40^−/−^IL-2Rα^−/−^* mice. Linear regression analysis between the ratio of B1a/B cells and the ratio of CD8^+^ T/T cells in PC **D.** and liver **E.** of *p40^−/−^IL-2Rα^−/−^* mice (*n* = 13).

Using correlation analysis, we noted that PC cell number was positively correlated with the number of liver MNCs (*P* = 0.0120, Figure 2A) in *p40^−/−^IL-2Rα^−/−^* mice, and the frequency of B1a in B cells was negatively correlated with PC and liver MNC numbers (*P* = 0.0300 and *P* = 0.0344, Figure 2B, 2C). In addition, there was a negative correlation between the frequency of B1a in B cells and the frequency of CD8^+^ T cells in PC and liver (*P* = 0.0030 and *P* = 0.0426, Figure 2D, 2E). Taken together, these data reflected that B1a cell population was negatively correlated with portal inflammation in *p40^−/−^IL-2Rα^−/−^* mice.

### Change of B1a cell population with age

**Figure 3:**
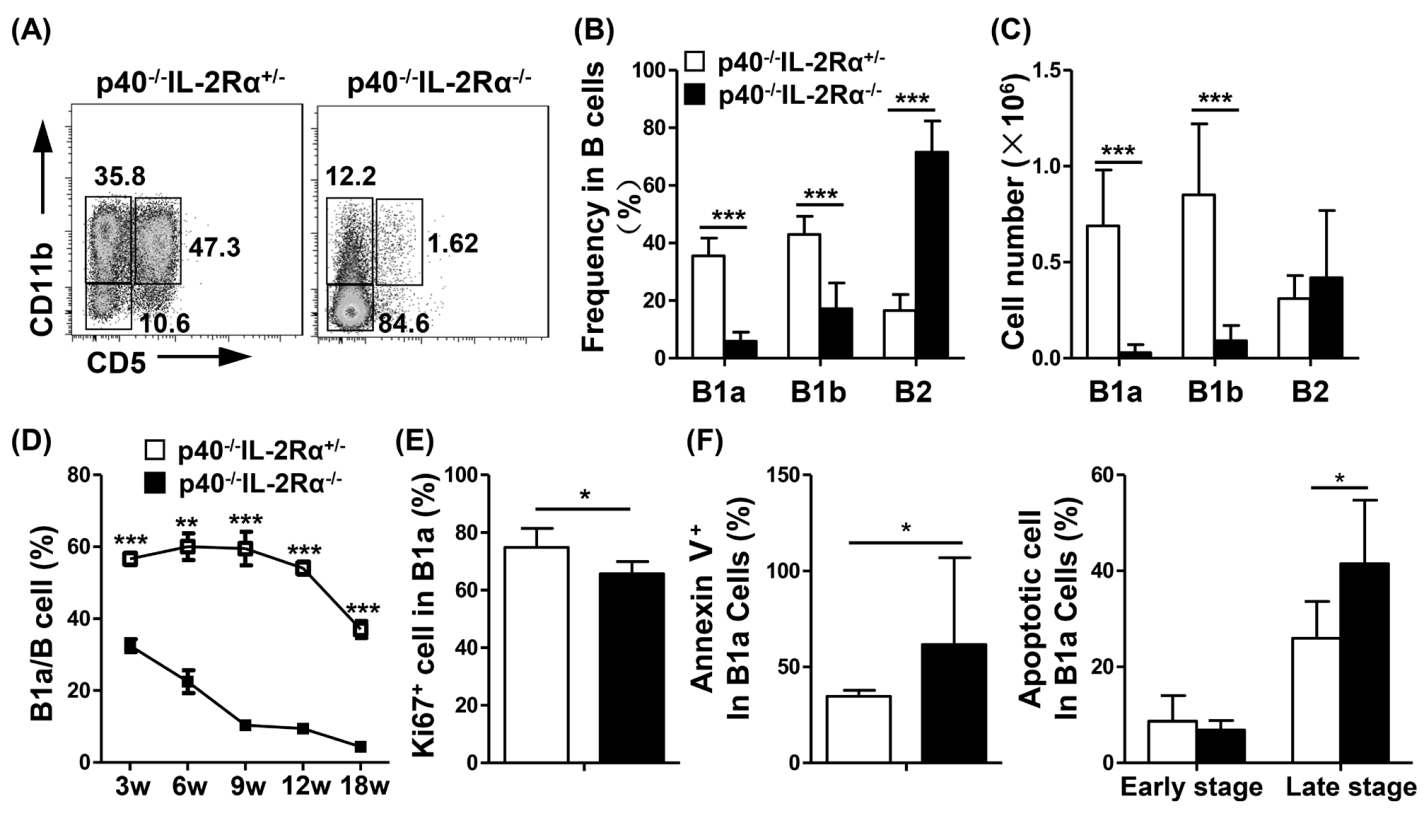
Comparison of B cell subpopulations in the PC of *p40^−/−^IL-2Rα^−/−^* mice and *p40^−/−^IL-2Rα^+/−^* mice **A.** A representative graph of PC B cell subsets at 18 weeks of age *p40^−/−^IL-2Rα^−/−^* mice and *p40^−/−^IL-2Rα^+/−^* mice. The frequency **B.** and cell number **C.** of B1a, B1b, and B2 cell subsets in the PC of *p40^−/−^IL-2Rα^−/−^* mice (*n* = 13) and *p40^−/−^IL-2Rα^+/−^* mice (*n* = 9). **D.** The frequency of B1a cells at different ages in PC of *p40^−/−^IL-2Rα^−/−^* mice and *p40^−/−^IL-2Rα^+/−^* mice (Each data point, n≥3). **E.** Ki67 expression in PC B1a cells from *p40^−/−^IL-2Rα^−/−^* (*n* = 6) and *p40^−/−^IL-2Rα^+/−^* mice (*n* = 6). **F.** The apoptosis level of PC B1a cells labeled with Annexin V and 7-AAD from *p40^−/−^IL-2Rα^−/−^* mice (*n* = 7) and *p40^−/−^IL-2Rα^+/−^* mice (*n* = 6).*P < 0.05, **P < 0.01, ***P < 0.001.

The frequency of B1a (CD11b^+^CD5^+^) (*P* < 0.0001) and B1b cells (CD11b^+^CD5^−^) (*P* < 0.0001) in B cells were much lower in *p40^−/−^IL-2Rα^−/−^* mice compared with *p40^−/−^IL-2Rα^+/−^* mice. B1a cells were almost undetectable, and the frequency of B2 (CD11b^−^CD5^−^) cells (*P* < 0.0001) in B cells were higher in PC from *p40^−/−^IL-2Rα^−/−^* mice compared to *p40^−/−^IL-2Rα^+/−^* mice (Figure 3A, 3B). We also detected another PBC mouse model, the *dnTGFβRII* mice, and found similar phenomenon that the frequency of B1a cells was decreased in PC of *dnTGFβRII* mice (*P* = 0.0238, Figure S3A). The numbers of B1a (*P* < 0.0001) and B1b cells (*P* < 0.0001) were markedly reduced in *p40^−/−^IL-2Rα^−/−^* mice compared with *p40^−/−^IL-2Rα^+/−^* mice, whereas the number of B2 cells (*P* = 0.3752) was not altered (Figure 3C). We also found the number of total B cells (*P* = 0.0214, Figure S2A) was decreased in spleen of *p40^−/−^IL-2Rα^−/−^* mice. But the frequency of B1a (*P* = 0.8113), B1b (*P* = 0.3832) and B2 (*P* = 0.5434) cells in spleen B cells did not change (Figure S2B). The frequency of B1a cells decreased gradually with age in *p40^−/−^IL-2Rα^−/−^* mice, and was always significantly lower when compared with that of *p40^−/−^IL-2Rα^+/−^* mice at the same age (*P* < 0.002, Figure 3D). B1a cells from *p40^−/−^IL-2Rα^−/−^* mice expressed lower levels of Ki67 (*P* = 0.0162, Figure 3E) and exhibited higher level of apoptosis (*P* = 0.0259, Figure 3F). The frequency of Annexin V^+^7-AAD^−^ early stage apoptotic B1a cells (*P* = 0.4089) did not change, but the frequency of Annexin V^+^7-AAD^+^ late stage apoptotic B1a cells (*P* = 0.0284) was significantly increased in the PC of *p40^−/−^IL-2Rα^−/−^* mice. These data demonstrated a positive correlation of the change in B1a cell frequency and disease progression in *p40^−/−^IL-2Rα^−/−^* mice.

### Reduction of expression of CTLA-4, GITR and IL-10 secretion in PC B cells of *p40^−/−^IL-2Rα^−/−^* mice

**Figure 4:**
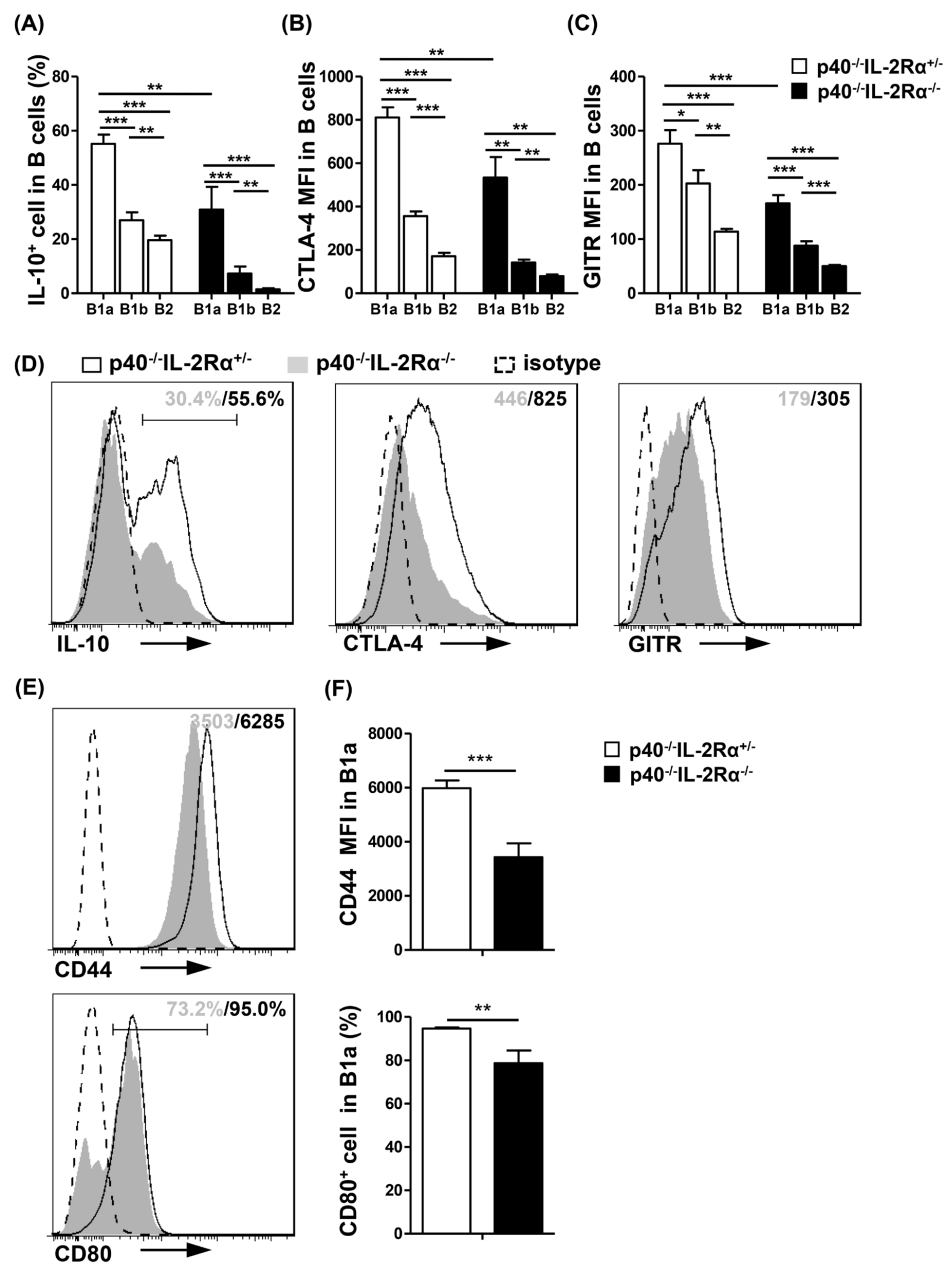
PC B cells from *p40^−/−^IL-2Rα^−/−^* mice displayed reduced regulatory and less activated phenotype Statistical analysis of frequency or mean fluorescence intensity (MFI) of regulatory molecules IL-10 **A.**, CTLA-4 **B.** and GITR **C.** in PC B cell subsets from *p40^−/−^IL-2Rα^−/−^* mice (*n* = 7) and *p40^−/−^IL-2Rα^+/−^* mice (*n* = 4). **D.** Expression of regulatory molecules (IL-10, CTLA-4, GITR) in PC B1a cells from *p40^−/−^IL-2Rα^−/−^* mice (*n* = 7) and *p40^−/−^IL-2Rα^+/−^* mice (*n* = 4). **E.** Expression of activation markers (CD44, CD80) in PC B1a cells from *p40^−/−^IL-2Rα^−/−^* mice (*n* = 4) and *p40^−/−^IL-2Rα^+/−^* mice (*n* = 3). **F.** Statistical analysis of frequency or mean fluorescence intensity (MFI) of phenotypic markers shown in **E.**. *P < 0.05, **P < 0.01, ***P < 0.001.

B1a cells had a much higher production of IL-10 (B1a *vs* B1b and B1a *vs* B2, *P* < 0.001, Figure 4A), and expression of CTLA-4 (B1a *vs* B1b and B1a *vs* B2, *P* < 0.001, Figure 4B) and GITR (B1a *vs* B1b, *P* = 0.0222; B1a *vs* B2, *P* < 0.001, Figure 4C) than B1b and B2 cells in *p40^−/−^IL-2Rα^+/−^* mice. Moreover, the secretion of IL-10, and expression of CTLA-4 and GITR were all significantly downregulated in *p40^−/−^IL-2Rα^−/−^* B1a cells compared with that of *p40^−/−^IL-2Rα^+/−^* mice (IL-10, *P* = 0.0041; CTLA-4, *P* = 0.0035; GITR, *P* < 0.001, Figure 4D). There were also distinct differences in B cell activation markers in B1a cells of *p40^−/−^IL-2Rα^−/−^* mice compared to *p40^−/−^IL-2Rα^+/−^* mice, including CD44 and CD80 (*P* = 0.0006 and *P* = 0.007, Figure 4E, 4F). Similar results were also detected in the PC B cell subsets of *dnTGFβRII* mice (Figure S3) These data suggested both qualitative and quantitative defects of B1a cells in the PC of *p40^−/−^IL-2Rα^−/−^* mice.

### PC B1a cells in *p40^−/−^IL-2Rα^−/−^* mice overexpress inhibitory markers and reduce regulatory markers

**Figure 5:**
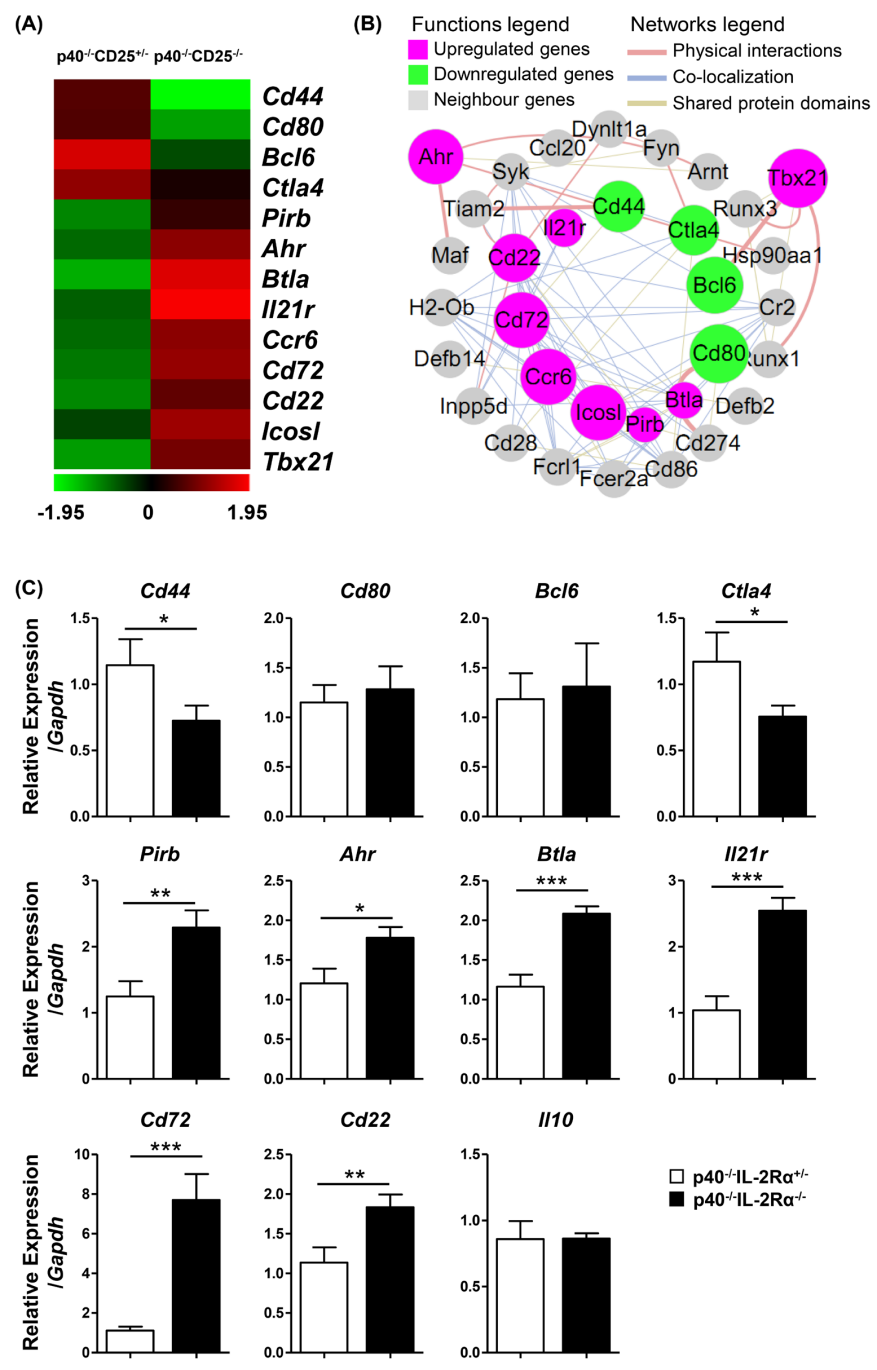
PC B1a cells of *p40^−/−^IL-2Rα^−/−^* mice exhibited altered regulatory characteristics compared to control B1a cells **A.** A heat map of selected genes from the transcriptional array were studied as noted in black (not significantly altered), green (down-regulated in *p40^−/−^IL-2Rα^−/−^* mice) or red (up-regulated in *p40^−/−^IL-2Rα^−/−^* mice). These genes were correlated with B cell function. **B.** Interaction network of these genes in **A.** and their first neighbor genes. Target genes were depicted as a circular node. Up-regulated genes are indicated in pink, while down-regulated genes are indicated in green of *p40^−/−^IL-2Rα^−/−^* mice B1a cells. The size of the circular node of B cell function genes were determined by the level of gene expression. **C.** Quantitative PCR analysis comparing mRNA expression level of selected genes between *p40^−/−^IL-2Rα^−/−^* and *p40^−/−^IL-2Rα^+/−^* mice. *P < 0.05, **P < 0.01, ***P < 0.001.

The B1a cell sorting purity was confirmed more than 96% (Figure S4). The microarray data (GEO accession number GSE79190) indicated that there was a differential transcription of 381 genes which changed greater than two fold in B1a of *p40^−/−^IL-2Rα^−/−^* mice. Amongst these 381 genes, 154 genes were up-regulated and 227 genes were down-regulated (Figure S5A), which included cytokine-cytokine receptor interactions, chemokine signaling pathways, transcription factor regulatory activity and cell cycle related genes (Figure S5B, S5C). Amongst B cell function related genes, in which the fold change were more than 1.3, *Cd44*, *Cd80*, *Bcl6* and *Ctla4* were down-regulated. In addition, the expression of *Ahr*, which could inhibit IL-10 secretion, was up-regulated. *Btla*, *Cd72* and *Cd22*, which could inhibit BCR signaling transduction through the ITIM (immunoreceptor tyrosine-based inhibitory motif) domain, was also up-regulated (Figure 5A). Moreover, the interaction network of these genes reflected that critical genes relevant to B cell function, including *Cd274 (Pdl1)*, *Cd28* and *Cd86*, were also closely related (Figure 5B). To confirm these data, we performed quantitative real-time PCR. We noted that *Cd44* and *Ctla4* were down-regulated. The expression of *Pirb*, *Ahr*, *Btla*, *Il21r*, *Cd72* and *Cd22* were significantly up-regulated (Figure 5C). Thus the quantitative PCR results confirmed our transcriptional microarray analysis.

## DISCUSSION

**Figure 6:**
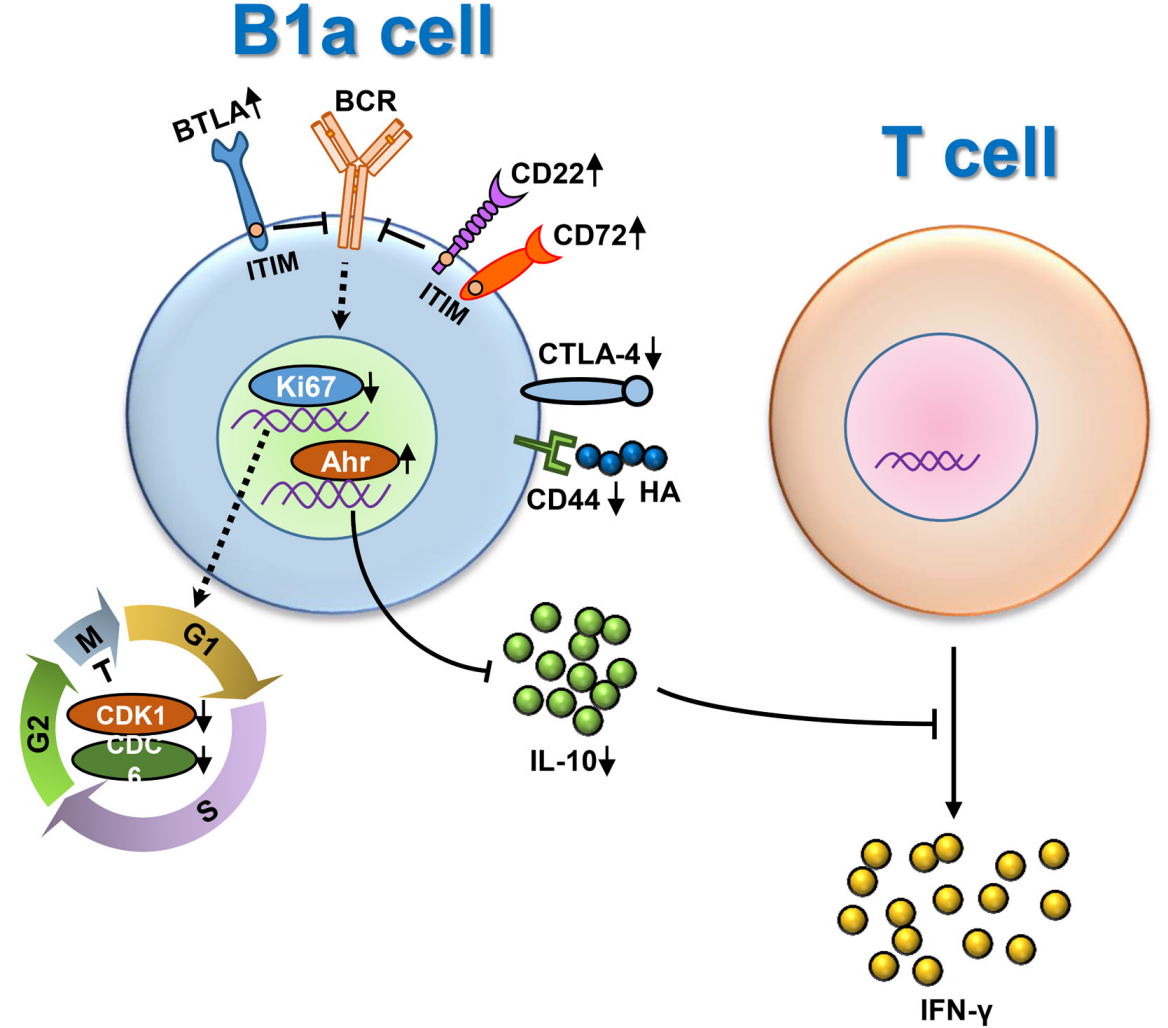
Dysregulation of peritoneal cavity B1a cells of *p40^−/−^IL-2Rα^−/−^* mice under inflammatory scenario On the one hand, some molecules with ITIM domains like BTLA, CD72 and CD22, were significantly increased to inhibit BCR signaling transduction. Meanwhile, the B1a cell cycle was delayed as the expression of Ki67 and other cell cycle related genes were decreased, leading to the reduced number of B1a cells. On the other hand, some regulatory molecules, e.g. IL-10, were significant decreased because of the up-regulation of Ahr. Consequently, the regulatory function of B1a cells was impaired, leading to the augmentation of IFN-γ secretion form T cells.

There has been significant attention on the role of B regulatory cells and loss of tolerance. In murine systems this has led to observations on peritoneal B1 cells [30, 34–36]. However, in humans, the role and definition of B1 cells is less clear and therefore the data herein on this murine model of autoimmune cholangitis is particularly relevant. Clearly B cell-related genes that impact function were altered with inflammation. For example, select molecules with ITIM domains such as BTLA, CD72 and CD22, were significantly increased as part of the inhibition of BCR signaling transduction. In contrast, some regulatory molecules, i.e. IL-10, were decreased because of the up-regulation of Ahr [37]. This discussion is also significant because of the relationship of the B1a cell cycle and the expression of Ki67 and its relationship to both qualitative and quantitative activity of B1a cells [38, 39]. Hence, our data herein reported the impairment of regulatory function and cell numbers of B1a cells in *p40^−/−^IL-2Rα^−/−^* mice, and its augmentation of T cell production of IFN-γ is significant (Figure 6).

Regulatory B cells are considered a new subset and have been shown to be integral to the maintenance of tolerance [40]. Indeed, B cells exert their regulatory role through the production of interleukin-10 (IL-10) by either B1 cells, marginal zone (MZ) B cells, or other B cell subpopulations [41–44]. IL-10 mediates suppression of inflammation by several mechanisms, including restricting the production of proinflammatory cytokines, such as IFN-γ and IL-17, downregulating the expression of MHC class II [40], and maintaining suppressive function of regulatory T cells [45]. Foxp3^+^ regulatory T cells derived IL-10 is important to restrain immune responses [46]. Foxp3^−^ T cells with IL-10 expression, i.e. Tr1 cells, also have regulatory abilities [47]. However, the suppressive function of regulatory B cells is primarily mediated by IL-10 [40] and thus the downregulated secretion of IL-10 by B1a cells in the PC of *p40^−/−^IL-2Rα^−/−^* mice has additional implications.

CTLA-4, an activation-induced homodimeric glycoprotein receptor, is expressed on T cells and interacts with the CD80/CD86 ligands which are also expressed on the surface of APCs and T cells [48, 49]. CTLA-4 acts as a negative regulator and functions to reduce T cell activation after B7 engagement, leading to down-regulation of T cell responses and to the preservation of T cell homeostasis and peripheral tolerance [50]. CTLA-4 is critical for the suppressive function of regulatory and conventional CD4^+^ T cells in both thymus and periphery [51–53]. It has been reported that B cells can express CTLA-4 in human and mice [54, 55], and this expression can be induced by activated T cells [56]. However, there is no significant data on the expression or function of CTLA-4 on PC B1a, B1b and B2 cells.

In our work the production of IL-10 and expression CTLA-4 were higher in B1a cells than B1b and B2 cells, suggesting that B1a cells may be regulatory B cells. In line with decreased B cell numbers, the immune regulatory function of PC B cells were impaired based on the expression of IL-10 and CTLA-4 in PC B cells in *p40^−/−^IL-2Rα^−/−^* mice. Hence, we hypothesized that in *p40^−/−^IL-2Rα^−/−^* mice, decreased number and function of regulatory B cells led to regulatory T cell dysregulation with subsequent increased inflammatory responses. B cell survival can be inhibited by CD4^+^ T cells [57], IFN-γ [58] and perhaps IL-21 [59, 60]. Therefore, an increase in Th1 and cytotoxic T cells in *p40^−/−^IL-2Rα^−/−^* mice may contribute to the decrease of B1a cells.

CD22 is a B cell transmembrane adhesion molecule with ITIMs in its cytoplasmic domain, and a negative regulator of B cell activation in response to local microenvironment [31]. CD22 inhibits BCR signaling by recruiting SHP-1, a protein tyrosine phosphatase, which can interfere with BCR-associated protein tyrosine kinase activation [61]. CD72 is also a B cell co-receptor containing ITIMs and also is an inhibitory co-receptor regulating the B cell immune response [62]. CD72*^−/−^* mice demonstrate increased B1a cell frequency in PC [63]. It is interesting that CD22 engagement before BCR stimulation can trigger the phosphorylation of CD72 [64]. These two molecules may have a synergistic role in regulating B cell activation. In our microarray and RT-PCR data, these same two molecules were both upregulated. Hence, there was significant inhibition of B1a cell activation in the PCs of *p40^−/−^IL-2Rα^−/−^* mice.

Under normal circumstances, IL-2Rα is expressed on regulatory and activated T cells; most lymphoid cells do not express IL-2Rα when directly examined *ex vivo* [65]. However, mature B cells can also express IL-2Rα. In mice, IL-2Rα can be expressed on B cells from PC [66]. Interestingly, murine CD25^+^ B cells have a higher expression of CD5, CD122 and CD132, suggesting that CD25^+^ B cells are B1a cells and can respond to IL-2. Murine CD25^+^ B cells, compared to humans, have a greater potential to secrete higher levels of IL-10 [67]. CD25^+^ B cells are detectable in humans and have more antigen-presenting ability than CD25^−^ B cells [68, 69]. CD25 can be upregulated in CD25^+^ B cells by Toll-like receptors, CD40 activation and IL-4 [68, 70]. In human, CD19^+^CD25^high^ B cells appear to function as immunoregulatory cells and are a source of inhibitory cytokines, including IL-10 and TGF-β [71]. However, it is not clear whether changes of B cell phenotype are due to an intrinsic deficiency of *IL-2Rα* or secondary to extrinsic factors such as an inflammatory microenvironment. We suggest that in the murine model herein, it is the microenvironment that leads to the dysregulation of PC B1a cells and therefore impairs their function, leading to a cascade and feedback loop which will further exacerbate pathology.

## MATERIALS AND METHODS

### Mice

*IL-2Rα^−/−^* (*B6.129S4-Il2ra^tm1Dw^*) and *p40^−/−^* (*B6.129S1-Il12b^tm1Jm^*) mice on a C57BL/6J background were initially obtained from The Jackson Laboratory (Bar Harbor, ME). *IL-2Rα^+/−^* mice were crossed with *p40^−/−^* mice to generate *p40^+/−^IL-2Rα^+/−^* mice, then backcrossed with *p40^−/−^* mice to generate *p40^−/−^IL-2Rα^+/−^* mice as breeding pairs. *IL-2Rα^+/−^* mice were used as breeding pairs to generate *IL-2Rα^+/−^* mice. We initially compared *p40^−/−^IL-2Rα^+/−^* mice to *IL-2Rα^+/−^* and control C57BL/6 (WT) mice and demonstrated that these strains were similar and healthy. Hence, *p40^−/−^IL-2Rα^+/−^* mice were used as controls throughout these studies. All mice were studied at 3-18 weeks and serially studied at 3, 6, 9, 12 and 18 weeks of age, with a minimum of 3 mice at each data point. Microarray and Real Time PCR assays were performed in 7-week old female mice. *dnTGFβRII* (*B6.Cg-Tg(Cd4-TGFBR2)16Flv/J*) mice, abbreviated as TG and used as another PBC mouse model [72], were obtained from the University of California at Davis, and studied at 12 weeks of age with littermate WT mice. All assays were performed in triplicate and each experiment replicated twice. All mice were maintained in individually ventilated cages under specific pathogen-free conditions at the Laboratory Animal Center, School of Life Sciences, University of Science and Technology of China. Animal experiments conformed to the guidelines outlined in the Guide for the Care and Use of Laboratory Animals by the Laboratory Animal Center, School of Life Sciences, University of Science and Technology of China.

### Liver histology

Liver tissues were excised and immediately fixed with 10% buffered formalin solution for 2 days at room temperature, then embedded in paraffin and cut into 4 μm sections for hematoxylin-eosin (H&E) staining [73]. Images were then obtained using an upright microscope (Axioskop 2 plus, Carl Zeiss, Oberkochen, Germany) in high-power fields (10×40).

### Isolation of hepatic and splenic mononuclear cells (MNCs) and peritoneal cavity cells

As previously outlined [74], we isolated hepatic MNCs as follows. Firstly, livers were disrupted using a syringe handle, suspended in PBS/0.2% BSA, and passed through a steel mesh. After centrifugation at 85g for one minute to remove the hepatocyte pellets, the residual cells were collected. MNCs from suspended liver cells were then isolated by centrifugation with 40% and 70% Percoll (GE Healthcare, Little Chalfont, United Kingdom) at 805g for 20 minutes, and cells at the interface were collected. Spleen was disrupted between two glass slides and red blood cells were lysed for 10 min. After neutralizing and centrifugation, pellets were resuspended with PBS. For peritoneal cavity cell isolation, the outer skin of the peritoneum was gently pulled back to expose the inner skin lining the peritoneal cavity. A needle was carefully inserted into the peritoneum to avoid puncture of organs [75]. 8 ml of ice cold PBS (with 2% BSA) was injected into the peritoneal cavity and washed repeatedly using a 10 ml syringe. The collected cell suspension was centrifuged at 453g for 5 minutes, the supernatant discarded and resuspended in media or PBS. Single cell suspensions were washed and counted. Viability of cells was confirmed by trypan blue exclusion.

### Collection of peritoneal cavity lavage fluid

A syringe containing 100ul ice cold PBS, with a 29G (0.33 mm) needle was carefully inserted into the peritoneum to avoid puncture of organs. After injection, the peritoneum was gently massaged for 2 minutes to dislodge any attached cells into the PBS solution. Thence an incision was made into the peritoneum and fluid collected with a Pasteur pipette; the fluid was then centrifuged at 5500g for 5 minutes and supernatant assayed using a cytometric bead assay (CBA) [10].

### Flow cytometry

1×10^6^ cell preparations were incubated with purified anti-CD16/CD32 antibody (BioLegend, San Diego, CA) for 15 minutes at 4°C. Cells were stained for 20 minutes at 4°C with cocktails containing combinations of fluorochrome-conjugated monoclonal antibodies for cell surface markers including Pacific Blue-CD3, Pacific Blue-PDCA-1, FITC-B220, APC/Cy7-CD4, APC/Cy7-IgD, APC/Cy7-CD19, PE/Cy7-NK1.1, PE/Cy7-CD23, PerCP/Cy5.5-IgM, APC-CD21/35, Alexa Fluor 647-CD11b, PE-F4/80, PE-CD5 (BioLegend), V500-CD4 and V500-CD8a (BD Biosciences, San Diego, CA). In PC, B cells were defined as CD19^+^CD3^−^. Gated on B cells, B1a cells were defined as CD11b^+^CD5^+^ and B1b cells were defined as CD11b^+^CD5^−^ whereas B2 cells were defined as CD11b^−^CD5^−^ [76]. In spleen, B cells were defined as B220^+^CD3^−^PDCA-1^−^. Gated on B cells, B1a cells were defined as CD23^−^CD5^+^IgM^hi^IgD^lo^ and B1b cells were defined as CD23^−^CD5^−^IgM^hi^IgD^lo^CD21/35^−^, whereas B2 cells, contained Follicular B cells (CD21/35^lo^CD23^+^) and Marginal zone B cells (CD21/35^hi^CD23^−^) [17]. The numbers of each cell subsets were calculated based on flow cytometric analysis of stained cells with specific fluorochrome-conjugated antibodies [77].

For intracellular cytokine staining, cells were suspended in RPMI-1640 with 10% fetal bovine serum (FBS) and stimulated with Cell Stimulation Cocktail (plus protein transport inhibitors) (eBioscience) at 37°C for 4 hours. Cells were incubated with purified anti-CD16/CD32 antibody and then stained with Pacific Blue-CD3, APC/Cy7-CD4, PerCP/Cy5.5-CD19, Alexa Fluor 647-CD11b, FITC-CD5, PE/Cy7-CD11b and PE/Cy7-NK1.1 (BioLegend), V500-CD8a (BD Biosciences) as above, fixed with fixation buffer, and permeabilized with permeabilization wash buffer (BioLegend); cells were thereafter stained for intracellular PE-IFN-γ, PE-IL-10, APC-IL-10, or PE-CTLA-4 (BioLegend). Normal IgG isotype controls (BioLegend) were used in parallel [78]. Intracellular Ki67 was identified by PE-Ki67 (BioLegend) using a Foxp3 staining kit (eBioscience).

Detection of apoptosis using a combined Annexin V and 7-AAD assay. 1×10^6^ cells were incubated with purified anti-CD16/CD32 antibody and then stained with FITC-CD4, PE-CD5, APC/Cy7-CD19 (BioLegend), and PE/Cy7-CD11b. The washed cells were resuspended in Annexin V binding buffer (BioLegend), and stained with Alexa Fluor 647-Annexin V (BioLegend) and 7-AAD (BioLegend) at room temperature for 15 min. Early stage apoptotic cells were defined as Annexin V^+^7-AAD^−^ and late stage apoptotic cells were defined as Annexin V^+^7-AAD^+^. FACSVerse flow cytometry (BD Immunocytometry Systems, San Jose, CA) was used to acquire data, which was analyzed by Flowjo software (Tree star, Inc., Ashland, OR).

### Gene-expression profiling analysis of peritoneal cavity B1a cells

PC B1a cells from 7-week old female *p40^−/−^IL-2Rα^−/−^* and *p40^−/−^IL-2Rα^+/−^* mice were isolated as above; the sorting purity was greater than 96%. Cells were suspended in TRIzol and total RNA extracted with an RNeasy Mini Kit (Qiagen, Hilden, Germany) and hybridized to Agilent chips. Fluorescence was detected using the Agilent Scanner G2505C (Agilent Technologies) and images were analyzed using Feature Extraction software (version 10.7.1.1, Agilent Technologies). A transcription profile chip service was provided by Shanghai OE Biotechnology Cooperation (Shanghai, China). Expression fluorescence values were log_2_-transformed, and subsequently analyzed using Genespring. Differentially expressed genes were defined as equal to or more than 2 fold differences between the two groups; signal values were confirmed beyond background signals, and the genes were classified based on the annotation of the Gene Ontology (GO) project. Volcano plot, heat map and red-green scale schemes were designed using Multiple Experiment Viewer 4.8 software. The microarray data was deposited on the National Center for Biotechnology Information GEO repository under accession number GSE79190.

### Prediction of target genes and the establishment of interaction networks

The target genes for all selected B cell functional genes were identified or predicted using three online bioinformatic databases, including GeneMANIA (http://www.genemania.org), STRING (http://string-db.org) and Gene Network Central (http://www.sabiosciences.com) [79]. The open source Bioinformatics software Cytoscape 3.2.1 (http://www.cytoscape.org) was used to create the interaction networks.

### Cytokines in PC lavage fluid

The levels of IFN-γ, TNF-α, IL-6, MCP-1 and IL-10 from PC lavage fluid were measured with a BD cytometric bead assay (CBA) “Mouse inflammation” kit (BD Pharmingen), using a BD FACSVerse flow cytometer with CBA software.

### Correlation analysis

Correlation analysis was made using GraphPad Prism 5 with linear regression analysis. The linear correlation index r^2^ and P values were calculated.

### Real time PCR

B1a peritoneal isolated cells were isolated from *p40^−/−^IL-2Rα^−/−^* and *p40^−/−^IL-2Rα^+/−^* mice; cell extraction was performed using Trizol Reagent (Invitrogen, Carlsbad, CA), and cDNA synthesized with the PrimeScript^®^ RT reagent Kit (Takara). Quantitative real-time PCR was performed by AB Stepone real-time PCR system (Applied Biosystems, Carlsbad, CA) and using SYBR^®^ Premix Ex TaqTM II (Takara). The primers used were noted in Table 2 and based on PrimerBank and confirmed using NCBI primer blast. The expression levels of target genes were normalized to the housekeeping gene *Gapdh* (ΔCt). The results were calculated by the 2^−ΔΔCt^ method.

**Table 2:** Real Time PCR primers used in this work

Genes	Forward primer 5′-3′	Reverse primer 5′-3′
*Cd44*	TCGATTTGAATGTAACCTGCCG	CAGTCCGGGAGATACTGTAGC
*Cd80*	ACCCCCAACATAACTGAGTCT	TTCCAACCAAGAGAAGCGAGG
*Bcl6*	CCGGCACGCTAGTGATGTT	TGTCTTATGGGCTCTAAACTGCT
*Ctla4*	TTTTGTAGCCCTGCTCACTCT	CTGAAGGTTGGGTCACCTGTA
*Pirb*	GACTTATGCCCAGGTGAAACC	AGATTCGGCAGCCTGATTGTT
*Ahr*	AGCCGGTGCAGAAAACAGTAA	AGGCGGTCTAACTCTGTGTTC
*Btla*	TGCTTGGGACTCCTCGGTTAT	ACACAGATTGTTCCATTGTGCT
*Il21r*	GGCTGCCTTACTCCTGCTG	TCATCTTGCCAGGTGAGACTG
*Cd72*	ATGGCTGACGCTATCACGTAT	CCTGTCCTAGATGGTTAGATGCG
*Cd22*	ATGCGCGTCCATTACCTGTG	TCAACGGTCCAATCATTTGCT
*Il10*	GCCAGAGCCACATGCTCCTA	GATAAGGCTTGGCAACCCAAGTAA

### Statistical analysis

Statistical significance was analyzed using GraphPad Prism 5 (GraphPad Software, San Diego, CA). All experiments were replicated at least three times with similar results. All data are expressed as mean ± standard deviation (SD), and evaluated using a 2-tailed unpaired Student t test.

## SUPPLEMENTARY MATERIALS FIGURES


