# Supplementary material for: Dysregulation of peritoneal cavity B1a cells and murine primary biliary cholangitis
Source: Oncotarget. 2016 Apr 20;7(19):26992–7006. doi: 10.18632/oncotarget.8853 (PMC5053627; doi:10.18632/oncotarget.8853)
Supplement: Supplementary file 1 [file oncotarget-07-26992-s001.pdf]

Dysregulation of peritoneal cavity B1a cells and murine primary biliary cholangitis

Supplementary Material

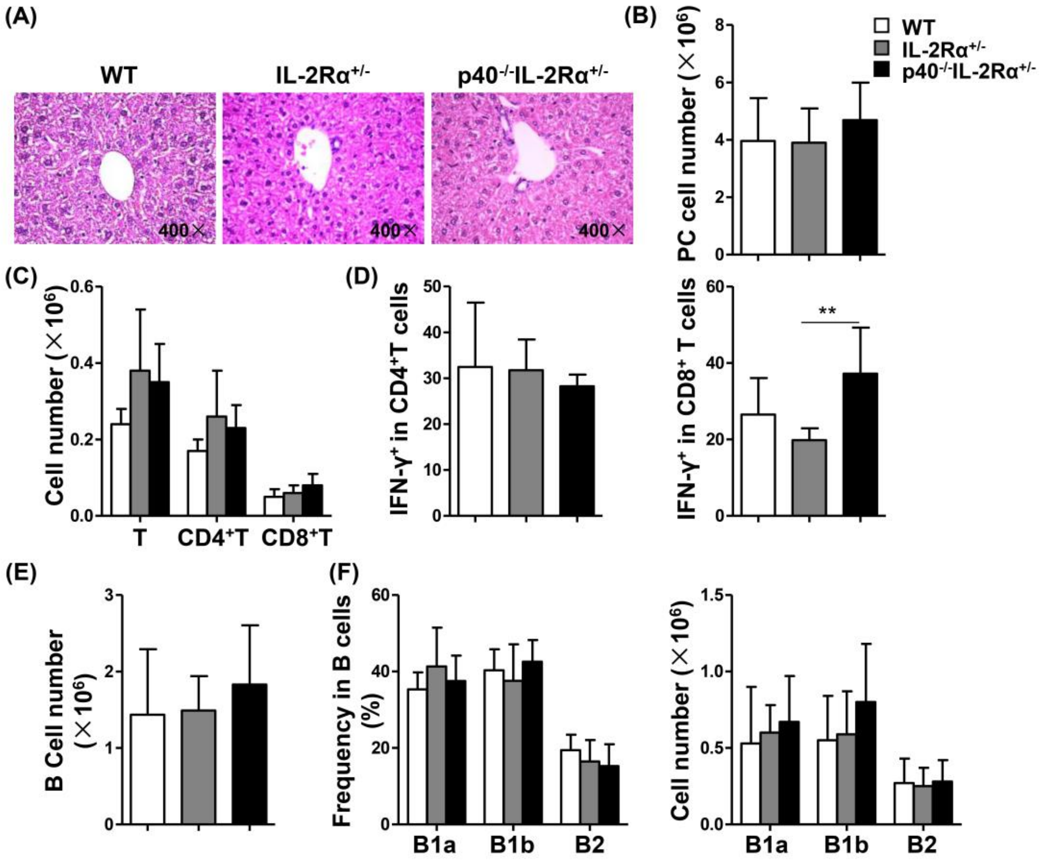

Supplementary Figure 1. Characteristics of PC cell subsets in the three control groups, including WT, *IL-2Rα*$^{+/-}$ and *p40*$^{-/-}$*IL-2Rα*$^{+/-}$ mice. (A) H&E staining of liver sections from WT, *IL-2Rα*$^{+/-}$ and *p40*$^{-/-}$*IL-2Rα*$^{+/-}$ mice. (B) Numbers of total cells in the PC of WT (n=7), *IL-2Rα*$^{+/-}$ (n=6) and *p40*$^{-/-}$*IL-2Rα*$^{+/-}$ mice (n=12). (C) Total number of T cells, CD4^{+} T, CD8^{+} T cells in the PC of WT (n=4), *IL-2Rα*$^{+/-}$ (n=6) and *p40*$^{-/-}$*IL-2Rα*$^{+/-}$ mice (n=4). (D) Frequency of IFN-γ^{+} cells gated on CD4^{+} and CD8^{+} T cells in PC of WT (n=3), *IL-2Rα*$^{+/-}$

(n=7) and *p40*$^{-/-}$*IL-2Rα*$^{+/-}$ mice (n=4). (E) Total number of B cells in the PC of WT (n=7), *IL-2Rα*$^{+/-}$ (n=6) and *p40*$^{-/-}$*IL-2Rα*$^{+/-}$ mice (n=12). (F) Frequency (left panel) and cell number (right panel) of B1a, B1b, and B2 cell subsets in the PC of WT (n=7), *IL-2Rα*$^{+/-}$ (n=6) and *p40*$^{-/-}$*IL-2Rα*$^{+/-}$ mice (n=12). *P < 0.05, **P < 0.01, ***P < 0.001.

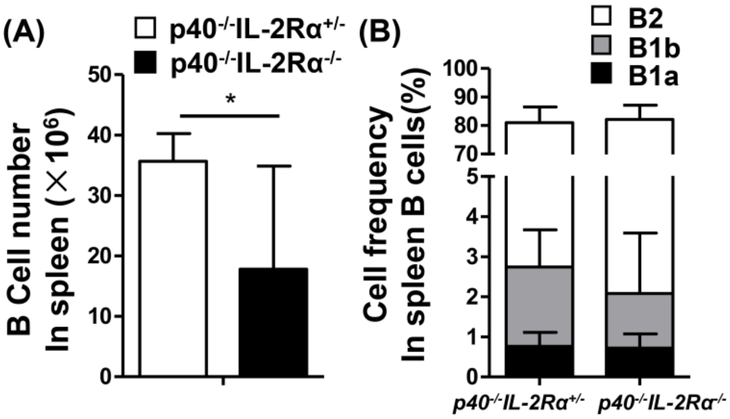

Supplementary Figure 2. The B cell number and phenotype in spleen of *p40*$^{-/-}$*IL-2Rα*$^{-/-}$ mice and *p40*$^{-/-}$*IL-2Rα*$^{+/-}$ mice. (A) The number of B cells in the spleen of *p40*$^{-/-}$*IL-2Rα*$^{-/-}$ mice (n=6) and *p40*$^{-/-}$*IL-2Rα*$^{+/-}$ mice (n=7). (B) The frequencies of B1a, B1b, and B2 cell subsets in the spleen B cells of *p40*$^{-/-}$*IL-2Rα*$^{-/-}$mice (n=6) and *p40*$^{-/-}$*IL-2Rα*$^{+/-}$mice (n=7). *P < 0.05, **P < 0.01, ***P < 0.001.

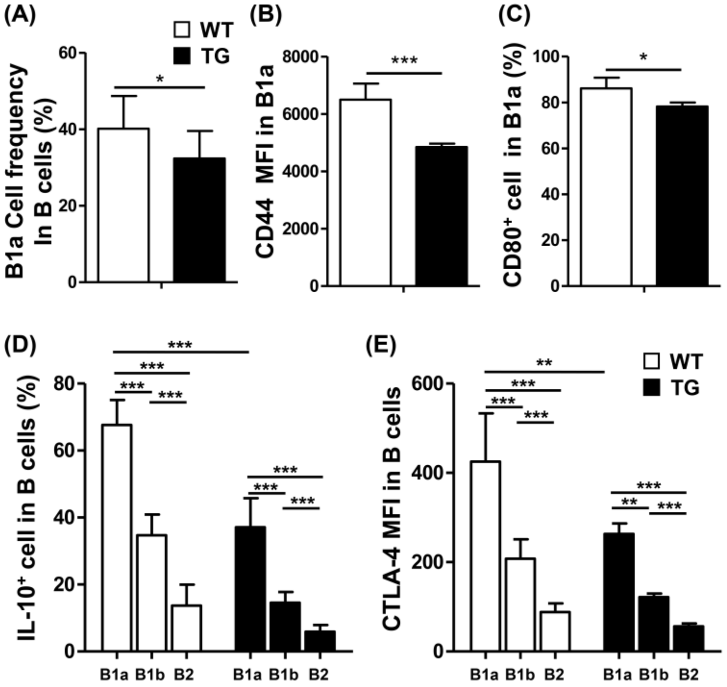

Supplementary Figure 3. The frequency and phenotype of PC B cells in *dnTGFβRⅡ* mice (TG). (A) The frequency of B1a cells in PC of *dnTGFβRⅡ* mice (n=11) and WT mice (n=14). Statistical analysis of mean fluorescence intensity (MFI) or frequency of CD44 (B), and CD80 (C) in PC B1a cells from *dnTGFβRⅡ* mice (n=5) and WT mice (n=5). Statistical analysis of frequency or MFI of regulatory molecules IL-10 (D), and CTLA-4 (E) in PC B cell subsets from *dnTGFβRⅡ* mice (n=5) and WT mice (n=5). *P < 0.05, **P < 0.01, ***P < 0.001.

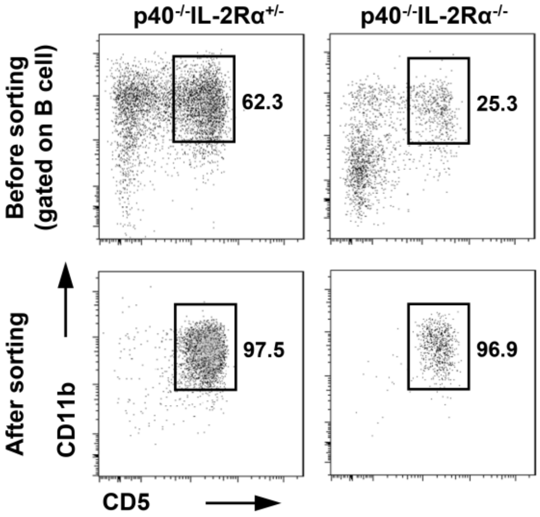

Supplementary Figure 4. The purity of PC derived B1a cells before and after sorting in *p40*$^{-/-}$*IL-2Rα*$^{-/-}$ and *p40*$^{-/-}$*IL-2Rα*$^{+/-}$ mice. Before sorting, cells were gated on B cells, and analyzed the expression of CD11b and CD5. After sorting, cells were harvested and expression of CD11b and CD5 was analyzed.

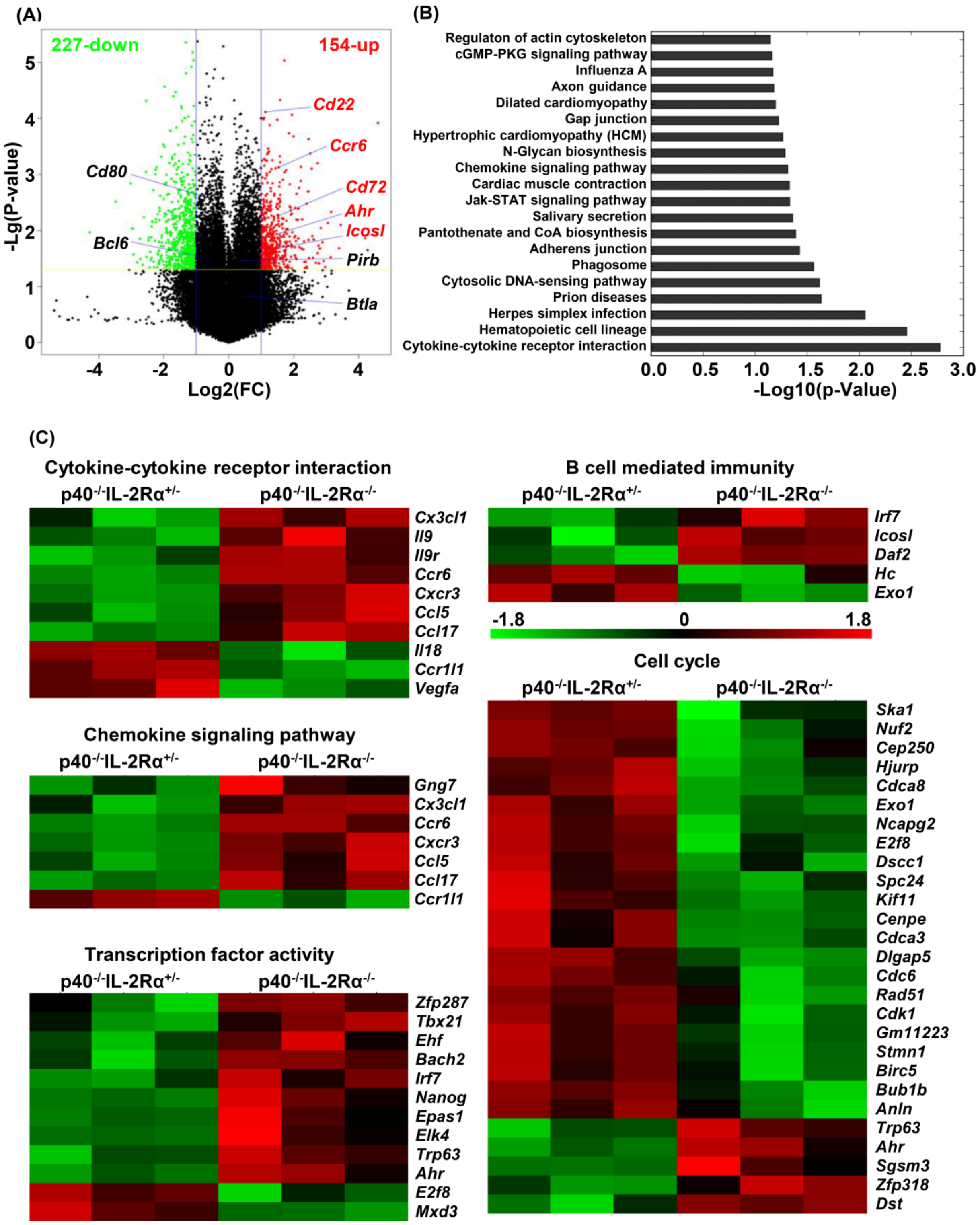

Supplementary Figure 5. Comparison of the transcription profile between B1a cells in the PC of *p40*$^{-/-}$*IL-2Rα*$^{-/-}$ and *p40*$^{-/-}$*IL-2Rα*$^{+/-}$ mice. (A) PC B1a cells were sorted as

CD19$^+$CD11b$^+$CD5$^+$ cells from 7 week old *p40$^{-/-}$IL-2Rα$^{-/-}$* and *p40$^{-/-}$IL-2Rα$^{+/-}$* mice. The gene expression profile from total RNA was examined by Agilent GeneChip analysis. Log_2 fold changes and their corresponding – Lg (P-value) of all genes in the microarray were used for construction of the volcano plot. Genes up-regulated more than two fold in PC B1a cells of *p40$^{-/-}$IL-2Rα$^{-/-}$* mice with a P-value ≤ 0.05 are depicted in red dots and those down-regulated with identical fold change and P-value are in green; all other genes in the array that were not significantly altered are shown in black. The yellow horizontal line represents: P-value = 0.05, -lg (P-value) = 1.3, the blue vertical lines represented gene fold change (FC) = 2 or 0.5, log_2 (FC) = ±1. All selected genes with a gene number were correlated with B cell function. (B) Kyoto Encyclopedia of Genes and Genomes (KEGG) pathway analysis of differentially expressed genes in PC B1a cells of *p40$^{-/-}$IL-2Rα$^{-/-}$* mice. P-value < 0.05 was used as a threshold to select significant KEGG pathways. The horizontal axis denotes the –Lg (P-value) of significance in pathways of *p40$^{-/-}$IL-2Rα$^{-/-}$* compared to *p40$^{-/-}$IL-2Rα$^{+/-}$* mice. (C) Heat maps representing the relative expression of genes among B1a samples from *p40$^{-/-}$IL-2Rα$^{-/-}$* and *p40$^{-/-}$IL-2Rα$^{+/-}$* mice. Genes that had more than a two-fold change with a P-value of ≤ 0.05 were sorted within each group. Expression statistics for each gene were centered, scaled and mapped to a color scale. Red represents relatively higher expression, whereas green represents relatively lower expression.
